# Feleucins: Novel Bombinin Precursor-Encoded Nonapeptide Amides from the Skin Secretion of *Bombina variegata*

**DOI:** 10.1155/2014/671362

**Published:** 2014-06-09

**Authors:** Bing Bai, Xiaojuan Hou, Lei Wang, Lilin Ge, Yu Luo, Chengbang Ma, Mei Zhou, Jinao Duan, Tianbao Chen, Chris Shaw

**Affiliations:** ^1^Natural Drug Discovery Group, School of Pharmacy, Queen's University, Belfast BT9 7BL, UK; ^2^Centre for Infection and Immunity, School of Medicine, Dentistry and Biomedical Sciences, Queen's University, Belfast BT9 7BL, UK; ^3^Jiangsu Key Laboratory for Traditional Chinese Medicine (TCM) Formulae Research, Nanjing University of Chinese Medicine, Nanjing 210046, China

## Abstract

The first amphibian skin antimicrobial peptide (AMP) to be identified was named bombinin, reflecting its origin from the skin of the European yellow-bellied toad (*Bombina variegata*). Bombinins and their related peptides, the bombinin Hs, were subsequently reported from other bombinid toads. Molecular cloning of bombinin-encoding cDNAs from skin found that bombinins and bombinin Hs were coencoded on the same precursor proteins. Here, we report the molecular cloning of two novel cDNAs from a skin secretion-derived cDNA library of *B. variegata* whose open-reading frames each encode a novel bombinin (GIGGALLNVGKVALKGLAKGLAEHFANamide) and a C-terminally located single copy of a novel nonapeptide (FLGLLGGLLamide or FLGLIGSLLamide). These novel nonapeptides were named feleucin-BV1 and feleucin-BV2, respectively. The novel bombinin exhibited 89% identity to homologues from the toads, *B. microdeladigitora* and *B. maxima*. The feleucins exhibited no identity with any amphibian AMP archived in databases. Synthetic feleucins exhibited a weak activity against *Staphylococcus aureus* (128–256 mg/L) but feleucin-BV1 exhibited a synergistic action with the novel bombinin. The present report clearly demonstrates that the skin secretions of bombinid toads continue to represent a source of peptides of novel structure that could provide templates for the design of therapeutics.

## 1. Introduction

Amphibians are generally slow moving, soft skinned and inhabit environments that are often laden with potentially pathogenic microbes [1]. Thus, in order to survive in such conditions, they have developed a potent chemical defense system within their skin secretions that contains, on the one hand, numerous pharmacologically active peptides to ward off predators and, on the other hand, a plethora of broad-spectrum antimicrobial peptides (AMPs) to prevent microbial infection [2]. In fact, the largest cohort of AMP structures currently known is from amphibian skin sources, despite their presence in many other life forms, including plants [3, 4].

Based on their primary structures and/or secondary structural characteristics, AMPs isolated from amphibian skin can be classified into three broad groups [4–6]. (1) The first contains the linear amphipathic helical peptides which are devoid of cysteinyl residues; examples are the magainins, isolated from the African clawed frog,* Xenopus laevis*, and the bombinins, isolated from bombinid toads. (2) The second group is the peptides isolated from various ranid frog species and these can be further classified into four different subgroups. However, all contain two cysteinyl residues that form an intramolecular disulphide bond at their C-terminals. (3) The third group includes the temporins, short peptides of usually 14 amino acid residues that were originally isolated from* Rana temporaria* [7]. Despite their diversity in primary structure, most AMPs contain a high proportion of both cationic and hydrophobic amino acid residues arranged to form amphipathic structures. This structural feature is a major determinant in facilitating their selective electrostatic interactions with the surfaces of bacterial membranes that are rich in anionic phospholipids [8–11].

AMPs were first reported from amphibian skin by Csordás and Michl in 1970, when they described the peptide bombinin, a 24-residue antimicrobial and haemolytic peptide from the skin of the European yellow-bellied toad,* Bombina variegata* [12]. This original report was somewhat underestimated in importance by the scientific community until the discovery of the magainins by Zasloff, in 1987, a paper that essentially launched the field of amphibian skin AMP research [13]. From this time, many amphibian species were studied and several groups revisited the bombinid toads, resulting in the discovery of a large number of peptides with antimicrobial activities [14]. These peptides were grouped into two families based on their primary structural features. (1) The bombinin-like peptides (BLPs) consisted of 24–27 amino acid residues with highly conserved amidated C-terminal sequences but with more variable sequences outside this region when compared to the prototype peptide, bombinin. (2) The bombinins Hs, which consist of 17–20 amino acid residues, are more hydrophobic and haemolytic. The “H” suffix was a reflection of their high degrees of both hydrophobicity and haemolytic activity. Members of each group have highly conserved overall structures differing from one another by only a few amino acid residues [14]. Many molecular cloning studies of skin peptide precursor-encoding cDNAs in bombinid toads have shown that bombinins and bombinin Hs are coencoded within the same biosynthetic precursors [15–17].

Here, we describe the molecular cloning of two novel peptide precursor-encoding cDNAs from a* Bombina variegata* skin secretion-derived cDNA library. Each encoded the same novel bombinin of 27 amino acid residues and also a second novel peptide of 9 amino acid residues with different primary structures in each precursor. The 9-residue peptides each possessed an N-terminal Phe (F) residue and a C-terminal amidated Leu (L) residue and were named feleucins, in accordance. Synthetic replicates of all three peptides (the bombinin and both feleucins) exhibited antimicrobial activity although the feleucins were significantly less potent than the bombinin. Nevertheless, the feleucins represent the prototypes of a new class of AMP from bombinid toad skin secretion and they are among the smallest endogenous AMPs so far reported from amphibian skin.

## 2. Materials and Methods

### 2.1. Specimen Biodata and Secretion Acquisition

Specimens of* B. variegata* (*n* = 12) were obtained from a commercial source as captive-bred metamorphs and were raised to maturity in vivaria for a period of 18 months. The skin secretions were obtained from the toads by gentle electrical stimulation (4 ms pulse width, 50 Hz, and 5 V), using platinum electrodes rubbed over the moistened dorsal skin surface at 10 s intervals following the procedure originally described by Tyler et al. [18]. Secretions were washed from the skin using deionised water, snap-frozen in liquid nitrogen, and lyophilised. All procedures on toads were carried out under appropriate UK animal licenses and had been approved by the local ethical committee. The sample used in the present study represented 15 mg dry weight of lyophilised skin secretion that had been dissolved in 5 mL of trifluoroacetic acid (TFA)/water (0.1 : 99.9, v/v) and clarified by centrifugation and the decanted supernatant was frozen at −20°C and stored in this state for 12 years [19]. A sample (0.5 mL) was originally removed for reverse phase HPLC analysis prior to freezing of the remainder. The frozen sample was removed from the freezer, thawed at room temperature, and then snap-frozen in liquid nitrogen prior to lyophilisation. Approximately, 12.5 mg dry weight of skin secretion residue was recovered following this procedure.

### 2.2. Molecular Cloning of Novel AMP Precursor-Encoding cDNAs

Five mg of lyophilised skin secretion was dissolved in 1 mL of mRNA stabilisation buffer and polyadenylated mRNA was isolated from this solution using magnetic oligo-dT beads as described by the manufacturer (Dynal Biotech, UK) and reverse-transcribed. The cDNA was subjected to 3′-RACE procedures to obtain full-length prepro-bombinin nucleic acid sequence data using a SMART-RACE kit (Clontech, UK) essentially as described by the manufacturer. Briefly, the 3′-RACE reactions employed an NUP primer (supplied with the kit) and a degenerate sense primer (S1; 5′-GATGAWKTTTAAGTACATARTTGCRGT-3′) (W = A/T; K = T/G; R = A/G) that was designed to a highly conserved domain in the 5′-untranslated region of previously characterised bombinin precursor-encoding cDNAs from* Bombina* species [15–17]. The PCR cycling procedure was as follows. Initial denaturation step: 60 s at 94°C; 35-cycle denaturation: 30 s at 94°C; primer annealing: 30 s at 58°C; extension: 180 s at 72°C. PCR products were gel-purified, cloned using a pGEM-T vector system (Promega Corporation), and sequenced using an ABI 3100 automated sequencer.

### 2.3. Identification and Structural Analysis of Novel AMPs Deduced from Cloned Precursor-Encoding cDNAs

A second 5 mg sample of lyophilised skin secretion was dissolved in 1 mL of 0.05 (v/v) trifluoroacetic acid (TFA)/water and clarified of microparticulates by centrifugation (1500 ×g) for 10 min. The clear supernatant was decanted and pumped directly onto a Cecil CE4200 Adept (Cambridge, UK) gradient reverse phase HPLC system, which is fitted with an analytical column (Phenomenex C-5, 0.46 cm × 25 cm). After a 10 min period of equilibration in start buffer (0.05% v/v TFA/water), peptides were eluted using a gradient formed from start buffer to 0.05/19.95/80.00 (v/v/v) TFA/water/acetonitrile in 240 min at a flow rate of 1 mL/min. Fractions were collected automatically at 1 min intervals and the column effluent was continuously monitored at *λ* 214 nm. Dead volume between column and fraction collector was minimal (20 *μ*L). The molecular masses of peptides in each chromatographic fraction were determined using matrix-assisted laser desorption/ionisation and time-of-flight mass spectrometry (MALDI-TOF MS) on a linear time-of-flight Voyager DE mass spectrometer (Perseptive Biosystems, MA, USA) in positive detection mode, using *α*-cyano-4-hydroxycinnamic acid as the matrix. Internal mass calibration of the instrument with known standards established the accuracy of mass determination as ±0.1%. Peptides with masses coincident with those of cloned precursor-deduced mature peptides were subjected to MS/MS fragmentation sequencing using an LCQ Fleet electrospray ion-trap mass spectrometer (Thermo Scientific, San Jose, CA, USA).

### 2.4. Solid-Phase Peptide Synthesis

Replicates of each of the three novel peptides were chemically synthesised by solid-phase Fmoc chemistry using a PS3 automated solid-phase synthesiser (Protein Technologies, Inc., AZ, USA). Following cleavage from the resin and deprotection, each synthetic peptide was analysed by both reverse phase HPLC and MALDI-TOF mass spectrometry to establish degree of purity and authenticity of structure.

### 2.5. Antimicrobial Assays

The antimicrobial activity of each synthetic peptide was assessed by means of determining minimal inhibitory concentrations (MICs) against reference strains of Gram-positive bacteria,* Staphylococcus aureus* (NCTC 10788), Gram-negative bacteria,* Escherichia coli* (NCTC 10418), and yeast,* Candida albicans* (NCPF 1467), respectively. The model microorganisms were initially incubated in Mueller-Hinton Broth (MHB) for 16–20 h. Upon achieving their respective logarithmic growth phases, as measured by the optical density (OD) of media at 550 nm, the cultures were diluted to 1 × 10^6^ colony-forming units (cfu)/mL for the bacteria and to 5 × 10^5^ cfu/mL for the yeast. Samples of these were then added to 96-well microtitre plates and mixed with the peptides in a range of concentrations between 1 and 512 mg/L. After a 24 h incubation, the OD of each well was measured at 550 nm using a Synergy HT plate reader (BioTek, USA), and the data were analysed using Graph Pad Prism 5 software. The MIC was defined as the minimum concentration of peptide which inhibits microbial growth (i.e., giving an OD identical to that of the negative control and culture medium with no organisms). Positive controls were also included and these contained organism cultures with no added peptides. After performing the MIC assays, 10 *μ*L of the medium from each well was inoculated onto a Mueller-Hinton agar (MHA) plate and incubated for 24 h for measurement of minimum bactericidal concentrations (MBCs). These were defined as the lowest concentration of peptide from which no colonies could be grown.

### 2.6. Assessment of Possible Synergistic Effects between AMPs

The possible synergistic interactions between the peptides were assessed by means of checkerboard titrations [20, 21]. In these assays, peptide A is diluted along the rows of a 96-well plate from concentrations of 4 × MIC to 0 × MIC, while peptide B is diluted in the same way along the columns. The tested microorganism used was the Gram-positive bacterium* S. aureus* (NCTC 10788). Peptide solutions of appropriate concentrations were inoculated with microorganism cultures (10^5^-10^6^ colony forming units/mL) and the 96-well plates were incubated for 18 h at 37°C in a humidified atmosphere. Growth was measured by optical density (OD) of the bacterial culture at *λ* 550 nm using an ELISA plate reader (BioTek Synergy HT). The result was calculated as the lowest cumulative fractional inhibitory concentration (∑FIC), where all wells were nonturbid but along the turbidity/nonturbidity interface. The calculation was as follows: ∑FIC = A/MIC_A_ + B/MIC_B_, where A and B represent respective MICs when in combination and MIC_A_ and MIC_B_ represent the MICs of peptide A and peptide B alone. The value of ∑FIC ≤ 0.5 indicates increasing degrees of synergy, whereas a ∑FIC of 1 shows an additive effect with no synergy [20, 21].

### 2.7. Haemolysis Assay

The haemolytic activity of each peptide was measured by incubating a range of concentrations of each synthetic peptide (1–512 mg/L) with a 4% suspension of horse erythrocytes that had been previously prepared by repeated washings with sterile PBS, centrifugations, and resuspensions. After incubation for 120 min, the suspensions were centrifuged at 900 ×g for 5 min to loosely pellet but not disrupt the cells. Optical density (OD) measurements of supernatants at 550 nm were recorded using a Synergy HT plate reader. The incubation of erythrocytes with 1% (v/v) Triton X-100 in PBS was designated as a positive control (100% haemolysis) and with PBS alone as a negative control (0% haemolysis).

## 3. Results

### 3.1. Molecular Cloning of Novel AMP Precursor-Encoding cDNAs

**Figure 1:**
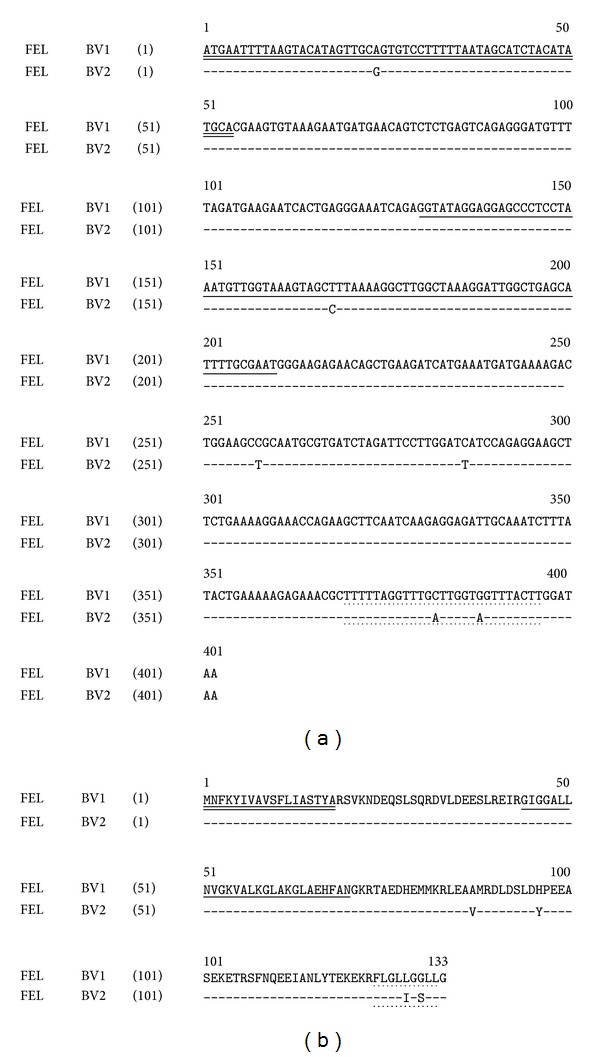
Alignment of open-reading frame nucleotide sequences of cloned cDNAs encoding feleucin-BV1 (FEL BV1) and feleucin-BV2 (FEL BV2) precursors. Putative signal peptide domains are double-underlined, mature bombinin domains are single-underlined, and mature feleucin domains are underlined with dotted lines. Sites of nucleotide sequence differences are indicated (a). Alignment of open-reading frame translated amino acid sequences of cloned cDNAs encoding feleucin-BV1 (FEL BV1) and feleucin-BV2 (FEL BV2) precursors. Putative signal peptide domains are double-underlined, mature bombinin domains are single-underlined, and mature feleucin domains are underlined with dotted lines. Sites of amino acid sequence differences are indicated (b).

Two different full-length cDNAs encoding novel peptides were consistently and repeatedly cloned (>25 replicates of each) from the skin secretion-derived cDNA library of* Bombina variegata* (Figure 1). Their respective open-reading frames exhibited very high degrees of similarity in both nucleotide and translated amino acid sequences (Figure 1) and each contained 133 amino acid residues. The domain architecture of each precursor protein was likewise identical consisting of a putative signal peptide (MNFKYIVAVSFLIASTYA) occupying residues 1–18, a single copy of the same novel 27-amino-acid-residue bombinin (GIGGALLNVGKVALKGLAKGLAEHFAN) occupying positions 44–70, and a different single copy of a novel 9-amino-acid-residue feleucin (BV1-FLGLLGGLL or BV2-FLGLIGSLL) occupying positions 124–132. The name feleucins was coined to phonetically represent their structural features of possessing N-terminal Phe (F) residues and C-terminal Leu (L) amide residues. Note that the two feleucins differ in primary structure by conservative substitutions at positions 5 (Leu/Ile) and 7 (Gly/Ser), respectively. Interestingly, bombinin is posttranslationally cleaved from the precursor by cleavage at a single Arg (R) residue flanking the N-terminal Gly (G) and it is cleaved and amidated following the C-terminal Asn (N) at a classical-GKR-cleavage/amidation site where the Gly (G) residue acts as a donor of its *α*-amino group to form the amide moiety. In contrast, the feleucins are cleaved from the precursor at a classical double basic cleavage site (-Lys-Arg-, -KR-) which flanks their respective N-terminals and are amidated directly by the Gly (G) residue amide donor that flanks their respective C-terminals. As in most amphibian skin peptide precursor proteins, the intervening peptide domains are rich in acidic amino acid residues.

The bombinin and feleucin amino acid sequences were separately subjected to online BLASTp (protein/protein) analyses, using the National Center for Biotechnology Information (NCBI) US database. Although the database contained many bombinins and related peptides, none were identical to the bombinin described here. The top hits were bombinins from the Oriental toads,* Bombina microdeladigitora* (accession number ABZ86151-89% identity),* Bombina maxima* (accession number Q58T59-89% identity), and* Bombina orientalis* (accession number CAC11122-85% identity). All of these bombinin precursors had typical bombinin H peptides encoded towards their C-terminals. In contrast, feleucin did not afford a single hit neither with any amphibian skin AMP peptide nor indeed with any AMP from any source. The nucleotide sequences of the precursor-encoding cDNAs of both peptides have been deposited in the EMBL Nucleotide Sequence Database under the accession codes HG794243 (feleucin-BV1) and HG794244 (feleucin-BV2).

### 3.2. Identification and Structural Analysis of Bombinin and Feleucins

**Figure 2:**
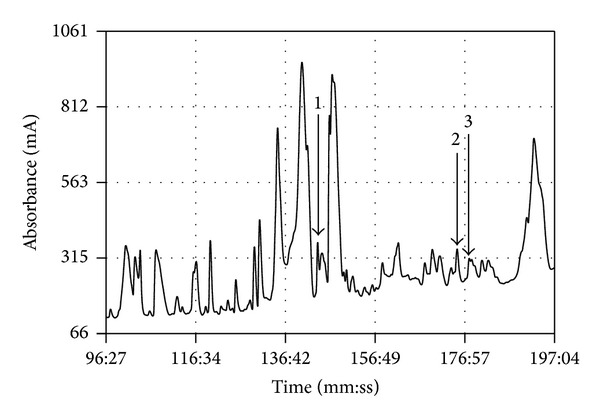
Region of reverse phase HPLC chromatogram of* Bombina variegata* skin secretion with arrows indicating elution positions/retention times of fractions containing peptides with molecular masses coincident with those predicted for the novel bombinin (arrow number 1), feleucin-BV2 (arrow number 2), and feleucin-BV1 (arrow number 3). The *y*-axis represents the absorbance in milliabsorbance units (mA) at *λ* 214 nm and the *x*-axis represents the retention time in minutes and seconds.

All three predicted novel peptides were identified in reverse phase HPLC fractions of* B. variegata* skin secretion based on their respective deduced monoisotopic molecular masses (bombinin = 2616.51 Da; feleucin-BV1 = 900.57 Da; feleucin-BV2 = 930.58 Da) as indicators in MALDI-TOF mass spectrometric analysis. Their elution positions/retention times are indicated in the appropriate regions of the reverse phase HPLC chromatogram in Figure 2. The fractions containing the predicted peptide molecular masses were infused into the electrospray ion-trap mass spectrometer and the appropriate doubly charged ions from each peptide were separately trapped and subjected to MS/MS fragmentation sequencing analysis to confirm their respective identities. The data obtained are shown in Table 1.

### 3.3. Antimicrobial and Haemolytic Assays

The data obtained in these assays are summarised in Table 2. The synthetic replicate of the novel bombinin, as expected, displayed relatively potent and broad-spectrum activity against all three test microorganisms with MICs of 8 mg/L, 32 mg/L, and 32 mg/L against* S. aureus*,* E. coli*, and* C. albicans*, respectively. The synthetic feleucins, in contrast, were only moderately active against* S. aureus* exhibiting MICs of 256 mg/L (BV1) and 128 mg/L (BV2), respectively. Both peptides had no effect on* E. coli* or* C. albicans* at concentrations up to and including 512 mg/L. The MBC values for the novel bombinin against all three test microorganisms were 32 mg/L and those for both feleucins against* S. aureus* were 256 mg/L (Table 2). The novel bombinin caused 100% haemolysis at a concentration of 64 mg/L while both feleucins were essentially ineffective in the lysis of red blood cells at the highest concentration tested (512 mg/L) (Table 2). The existence of possible synergistic effects between the novel bombinin and the feleucins was investigated using a checkerboard assay and the data are summarised in Table 3. The combination of feleucin-BV1 and feleucin-BV2 produced an additive effect (FIC index of 1.0) as did the combination of the novel bombinin and feleucin-BV2 (FIC index of 0.625). However, the novel bombinin and feleucin-BV1 in combination produced a synergistic effect (FIC index of 0.5).

## 4. Discussion

AMPs constitute the most abundant group of peptides in amphibian skin secretions and occur invariably alongside an array of pharmacologically active peptides (PAPs) rendering these secretions among the most molecularly diverse in nature [1–9, 22]. Their primary functions are in chemical defense against predators (PAPs) and in preventing infection from microorganisms in their microbially rich environments (AMPs) [1–9]. Many families of AMPS have been reported from such secretions and members of each are classified on the basis of their primary structural similarities and/or the presence of well-defined structural motifs [1–9].

One of the archetypal families of amphibian skin AMPs is the bombinins, originally discovered in the skin of* Bombina variegata* due to their haemolytic/antimicrobial activities [12]. Bombinins have subsequently been found in abundance and with a high degree of primary structural diversity in the skins/skin secretions of all bombinid toads investigated to date [14]. In addition, within a single species, the bombinins occur in multiple isoforms often differing by as little as a single amino acid residue and they are generally C-terminally amidated [14].

Following the advent of molecular cloning technology and its application to amphibian skin peptide research, the cDNAs encoding the biosynthetic precursors of bombinins were successfully cloned. Each translated amino acid sequence was found to contain both single copies of a bombinin and, located towards the C-terminus, a single copy of a bombinin H [15–17]. The latter peptides were so named as they possessed a more potent haemolytic activity than bombinins due to their higher content of hydrophobic amino acid residues [14].

Here, we have cloned two novel cDNAs from a skin secretion-derived library of* B. variegata*, one of the most extensively and longest-studied species; and while each encodes a precursor containing a single copy of a novel bombinin, in each there is a single copy of a different novel nonapeptide amide located towards their C-terminals. These peptides were named feleucins-BV1 and BV2, and they differ in primary structure by two conservative substitutions, L/I at position 5 and G/S at position 7. Of their nine amino acid residues, 6 are hydrophobic (F/L/I) and 3 are hydrophilic (G/S). Neither of the feleucins contained an acidic (E/D) or basic (K/R) amino acid residue and their C-terminal carboxyl groups were blocked through amidation. Despite their high degree of hydrophobicity, neither peptide possessed haemolytic activity up to concentrations of 512 mg/L, in contrast to the bombinin H peptides [23]. However, their antimicrobial potencies were relatively low and both were only effective against the model Gram-positive bacterium* S. aureus*. It remains unclear whether AMPs in these complex defensive skin secretions act on their own or cooperate/interact with others. Early studies on AMPs from African clawed frog (*Xenopus laevis*) skin suggested that cooperative effects did in fact occur between different peptides [24]. In view of this, it was decided to investigate possible synergistic actions between the novel bombinin and the feleucins using a standard checkerboard assay [20, 21]. Bombinin in combination with feleucin-BV2 and both feleucins in combination produced additive effects with no evidence of synergism. In contrast, bombinin in combination with feleucin-BV1 produced a clear synergistic effect. In view of the high degree of structural diversity of AMPs in amphibian defensive skin secretions and the widely held view that such secretions, like venoms, act in a holistic manner through numerous intermolecular interactions, it would not be surprising to observe such cooperative effects and, indeed, this has been previously reported for the magainins from* Xenopus* skin [1–9, 24]. Although the feleucins are among the smallest amphibian skin AMPs, recently, an AMP consisting of 8 amino acid residues—FFFLSRIFa—and named temporin-SHf was reported from the skin of the North African ranid frog,* Pelophylax saharica *[25]. While this AMP had a more potent and broad-spectrum activity than the feleucins, this was probably due to its high content of phenylalanyl (F) residues (50%) and the possession of a cationic residue (arginine-R). Enhancing the cationicity of the feleucin template may result in parallel enhancement of potency and spectrum of action.

## 5. Conclusions

In conclusion, novel prototype AMPs, the feleucins, have been identified in the skin secretion of the bombinid toad,* B. variegata*, and are coencoded in a common precursor protein with a novel bombinin. These nonapeptide amides are among the smallest AMPs so far reported from amphibian skin secretions and are, additionally, among the smallest AMPs so far reported from nature. Amphibian skin secretions, even those of intensively studied species, such as* B. variegata*, still represent unique resources for the discovery of novel bioactive peptides that could serve as templates for the rational design of novel peptide therapeutics.

## Figures and Tables

**Table:** (a)

Number 1	*b*(1+)	*b*(2+)	Seq.	*y*(1+)	*y*(2+)	Number 2
1	58.02875	29.51801	G			27
2	171.11282	86.06005	I	2560.50874	**1280.75801**	26
3	228.13429	114.57078	G	2447.42467	**1224.21597**	25
4	**285.15576**	143.08152	G	2390.40320	**1195.70524**	24
5	**356.19288**	178.60008	A	2333.38173	**1167.19450**	23
6	**469.27695**	235.14211	L	2262.34461	**1131.67594**	22
7	**582.36102**	291.68415	L	2149.26054	**1075.13391**	21
8	**696.40395**	**348.70561**	N	2036.17647	**1018.59187**	20
9	**795.47237**	**398.23982**	V	1922.13354	**961.57041**	19
10	**852.49384**	**426.75056**	G	1823.06512	**912.03620**	18
11	**980.58881**	**490.79804**	K	1766.04365	**883.52546**	17
12	**1079.65723**	**540.33225**	V	**1637.94868**	**819.47798**	16
13	**1150.69435**	**575.85081**	A	**1538.88026**	**769.94377**	15
14	**1263.77842**	**632.39285**	L	**1467.84314**	**734.42521**	14
15	**1391.87339**	**696.44033**	K	**1354.75907**	**677.88317**	13
16	1448.89486	**724.95107**	G	**1226.66410**	**613.83569**	12
17	**1561.97893**	**781.49310**	L	**1169.64263**	585.32495	11
18	**1633.01605**	**817.01166**	A	**1056.55856**	528.78292	10
19	1761.11102	**881.05915**	K	**985.52144**	**493.26436**	9
20	1818.13249	**909.56988**	G	**857.42647**	**429.21687**	8
21	1931.21656	**966.11192**	L	**800.40500**	400.70614	7
22	2002.25368	**1001.63048**	A	**687.32093**	344.16410	6
23	2131.29628	**1066.15178**	E	**616.28381**	**308.64554**	5
24	2268.35519	**1134.68123**	H	**487.24121**	244.12424	4
25	2415.42361	**1208.21544**	F	350.18230	175.59479	3
26	2486.46073	**1243.73400**	A	203.11388	102.06058	2
27			N-Amidated	132.07676	66.54202	1

**Table:** (b)

Number 1	*b*(1+)	Seq.	*y*(1+)	Number 2
1	148.07570	F		9
2	261.15977	L	**754.51859**	8
3	318.18124	G	**641.43452**	7
4	**431.26531**	L	**584.41305**	6
5	**544.34938**	L	**471.32898**	5
6	**601.37085**	G	358.24491	4
7	**658.39232**	G	**301.22344**	3
8	**771.47639**	L	244.20197	2
9		L-Amidated	131.11790	1

**Table:** (c)

Number 1	*b*(1+)	Seq.	*y*(1+)	Number 2
1	148.07570	F		9
2	**261.15977**	L	**784.52915**	8
3	**318.18124**	G	671.44508	7
4	**431.26531**	L	**614.42361**	6
5	**544.34938**	I	**501.33954**	5
6	**601.37085**	G	**388.25547**	4
7	**688.40288**	S	331.23400	3
8	**801.48695**	L	**244.20197**	2
9		L-Amidated	**131.11790**	1

**Table 2:** Summary of the minimum inhibitory concentrations (MICs) and minimum bactericidal concentrations (MBCs) of the three novel AMPs (bombinin, feleucin-BV1, and feleucin-BV2) described in this study. NA: not active. Haemolysis values are those effecting 100% lysis of red blood cells in 120 min.

Peptides	MIC (mg/L)			MBC (mg/L)		Haemolysis (mg/L)	
	*S. aureus *	*E. coli *	*C. albicans *	*S. aureus *	*E. coli *	*C. albicans *	Horse red cells
Bombinin	8	32	32	32	32	32	64
Feleucin-BV1	256	NA	NA	256	NA	NA	>512
Feleucin-BV2	128	NA	NA	256	NA	NA	>512

**Table:** (a)

Peptide combination	FIC index	Result of combination
Feleucin-BV1 + feleucin-BV2	1	Additive
Bombinin + feleucin-BV1	0.5	Synergistic
Bombinin + feleucin-BV2	0.625	Additive

**Table:** (b)

Microorganism	Lowest FIC index ([A]/[B] in mg/L)		
	Peptide A + peptide B	Peptide A + peptide C	Peptide B + peptide C
*S. aureus *	0.5 (2/64)	0.625 (4/16)	1 (128/64)

Peptide A is bombinin.

Peptide B is feleucin-BV1.

Peptide C is feleucin-BV2.

[A] and [B] are the MIC fractions or multiples in combination.
